# Alpaca Keeping in Hungary: The First Nationwide Survey

**DOI:** 10.3390/ani16081209

**Published:** 2026-04-16

**Authors:** Viktória Láng, András Adorján, Viktor Jurkovich

**Affiliations:** 1 Centre for Animal Welfare, University of Veterinary Medicine, 1078 Budapest, Hungary; 2 András Adorján Mobile Veterinary Practice, 2117 Isaszeg, Hungary

**Keywords:** alpaca, camelids, animal husbandry, livestock management, animal health, biosecurity, veterinary care, survey study, Hungary

## Abstract

Alpacas have become increasingly popular as farm and companion animals in many countries and were introduced in Hungary approximately 15 years ago. However, reliable data on their population size and management practices have remained limited. Therefore, this study aimed to provide the first nationwide overview of alpaca keeping in Hungary. An anonymous Google Forms-based questionnaire comprising 55 questions was used to collect data on farm characteristics, herd size, husbandry practices, and animal health management. A total of 53 valid responses were analyzed. The results indicated that approximately 260 alpacas are currently kept in Hungary, with most herds being small and typically consisting of 1–5 animals. Most owners planned to expand their herds, primarily through domestic breeding or purchases; however, only a minority considered alpaca keeping economically profitable. Responses related to quarantine, veterinary access, and disease management suggested limited knowledge of biosecurity and animal health. While most owners reported high levels of trust in veterinarians, many had difficulty accessing specialists with expertise in alpacas. In conclusion, alpaca keeping in Hungary is a growing but still small-scale sector. Improving owner education and strengthening professional support may enhance animal welfare and reduce health risks, thereby supporting the One Health approach.

## Simple Summary

Alpacas have become increasingly popular as farm and companion animals in Hungary during the past decade. However, little reliable information is available on how many alpacas are kept, how they are managed, and the health challenges their owners face. In this study, we carried out the first nationwide survey on alpaca keeping in Hungary using an anonymous online questionnaire. Our results show that most herds are small and spread across the country, and that many owners plan to increase their herd sizes. Although most breeders trust veterinarians and seek professional advice, many report difficulties in finding specialists with experience in alpaca medicine. In addition, knowledge about disease prevention and hygiene is often limited. Improving owner education and making reliable information more accessible may help protect animal health, improve animal welfare, and reduce health risks for both animals and people.

## 1. Introduction

The alpaca (*Vicugna pacos*) is generally considered to have been domesticated primarily from the South American vicuña (*Vicugna vicugna*) in the high-altitude regions of the Andes more than 5000 years ago, with evidence of genetic introgression from llamas [1,2]. This process is considered one of the most important economic and cultural achievements of Andean civilizations, as alpaca fiber was used for clothing, blankets, and ceremonial garments for centuries [3]. Historically, alpacas were primarily used for fiber production under harsh mountain conditions, while llamas mainly performed pack transport. Their adaptability, stress tolerance, and low nutritional requirements enabled successful breeding at high altitudes (3500–5000 m above sea level), under conditions characterized by low oxygen levels, high solar radiation, and large temperature fluctuations. Alpaca fiber possesses exceptional physical properties: it is extremely fine, soft, highly insulating, and does not contain lanolin, and is therefore generally considered less allergenic compared to wool [4,5]. Alpaca fiber typically has a diameter of approximately 18 to 30 microns, depending on the type and quality. Textiles made from alpaca fiber, including clothing, yarn, and blankets, represent valuable raw materials in the international luxury industry, as confirmed by recent studies [6]. Two main alpaca types, Huacaya and Suri, can be distinguished based on fiber characteristics and appearance. Huacaya alpacas have soft, dense, and crimped, sheep-like fleece, whereas Suri alpacas produce long, silky fibers that hang in locks.

The global alpaca population was estimated at approximately 4–5 million animals in recent years, with Peru accounting for the largest share, with around 85% of the world’s total [7]. Bolivia hosts approximately 9–10% of the global population, with the remaining animals distributed across Chile, Argentina, and other countries.

Systematic commercial imports of alpacas into Europe began in the early 1990s, primarily in the UK, with animals sourced mainly from Chile and later from Peru. From the mid-1990s onward, alpaca keeping expanded rapidly across continental Europe. Today, alpacas are present in a wide range of European countries, although population sizes vary considerably and are not always supported by official registry data [8,9,10,11]. The UK hosts the largest European population, with estimates ranging from 35,000 to 45,000 registered animals [8,9]. Germany has become the largest continental European hub, with an estimated population of approximately 20,000 animals, and has been the subject of the most extensive research on European alpaca keeping [10]. Huacaya alpacas are more widespread, likely due to their easier management and shearing, as well as their suitability for temperate conditions.

While fiber and breeding stock production remain dominant in South America [7] and Australia [12], the growth of alpaca keeping in European countries has been driven primarily by hobby, recreational, and therapeutic purposes, as well as fiber production [13,14,15]. The welfare of alpacas is closely linked to service quality and sustainability in animal-assisted services, education, and ecotourism programs [15]. Within the European Union, animal welfare in such contexts is regulated primarily by general animal welfare legislation, as no species-specific regulations exist for camelids in most countries. The increasing use of alpacas in tourism and animal-assisted services may pose potential welfare risks, including excessive human interaction, inadequate handling practices, and insufficient knowledge of species-specific needs. Therefore, alpaca keeping represents not only an economic but also an ethical issue. Appropriate housing conditions, opportunities for social interaction, low-stress handling, and regular veterinary supervision are fundamental welfare requirements [16,17]. Studies indicate that health and behavioral problems in alpacas are often directly related to housing conditions and stress sensitivity [15,17,18].

In Hungary, the history of alpaca breeding dates back to approximately 2008 [19,20]. The first privately kept alpacas appeared mainly as hobby animals. In recent years, however, the species’ popularity has increased rapidly, and several small and medium-sized herds are now present across different regions of the country. At the same time, reliable official data on the number of alpaca herds and animals in Hungary is not available. Alpacas are not included in the national livestock identification system, and registration is not mandatory so far; therefore, population estimates rely primarily on voluntary records or informal sources. This lack of centralized data underscores the need for a systematic assessment of alpaca-keeping practices in Hungary.

The present study aims to provide a descriptive overview of alpaca keeping in Hungary and to address the lack of reliable baseline data on herd numbers, management practices, and animal welfare conditions.

## 2. Materials and Methods

### 2.1. Data Collection and Questionnaire Design

Data collection was conducted between May and July 2023 using an anonymous online questionnaire (Supplementary File). The questionnaire design was based on previous surveys, including studies conducted in Germany [10] and the UK [8] that examined husbandry and welfare practices in camelids.

The questionnaire consisted of 55 closed- and open-ended questions organized into three main sections: (i) owner-related information, including age, gender, previous experience with animal keeping, and herd size and type; (ii) housing and management conditions, including herd composition, breeding practices, feeding, grazing, housing systems, and co-housed species; and (iii) animal health and welfare aspects, including observation frequency, parasite control practices, access to veterinary services, record-keeping routines, and self-assessment of welfare-related knowledge. The questionnaire included single-choice, multiple-choice, short-answer, and linear-scale questions. Linear scales were used, for example, to assess perceptions of economic viability (1 = unfavorable, 10 = profitable), thereby capturing subjective experiences and motivation. Animal welfare aspects were further explored in the third section, including daily observation frequency, disease prevention, veterinary involvement, and social interactions.

Many respondents were members of alpaca-breeder communities; therefore, the questionnaire was distributed primarily through Facebook breeder groups and professional networks, with no additional formal distribution channels used.

The questionnaire was not formally pilot-tested or subjected to formal validation or reliability assessment; however, it was reviewed before distribution to ensure clarity and relevance. In particular, it was evaluated by an experienced alpaca keeper with practical expertise, and informal feedback was also obtained from members of alpaca breeder communities via online platforms. Minor adjustments were made based on these inputs before the final distribution.

Due to the lack of official data on the total number of alpaca breeders in Hungary and the absence of a centralized registry, the response rate could not be calculated, and the representativeness of the sample cannot be fully determined. Furthermore, as the questionnaire was distributed primarily through online breeder communities, this approach may have introduced selection bias toward more active and engaged respondents.

During the preparation of this manuscript, the authors used Claude AI (3.5 Sonnet) for the purposes of targeted search of the literature, and Grammarly.com to correct grammatical and spelling errors. The authors have reviewed and edited the output and take full responsibility for the content of this publication.

### 2.2. Data Processing and Statistical Analysis

Survey responses were recorded and analyzed using descriptive statistical methods. As the study was exploratory in nature and baseline data were limited, the analysis was intentionally restricted to descriptive statistics. Incomplete responses were excluded from the analysis, and only fully completed questionnaires were included in the final dataset. Missing data were not imputed. Data processing and analysis were conducted using Microsoft Excel, which was considered appropriate for the descriptive scope of the study. Frequencies and proportions of key variables were calculated and expressed as percentages. Anonymous identifiers were assigned to respondents to ensure that no personal data could be traced during analysis.

For welfare-related assessment, responses were grouped into the following categories: frequency of health monitoring (daily, weekly, monthly, never); access to veterinary services (local, distant, unavailable); frequency and type of antiparasitic treatment; access to exercise areas and enclosures (open, semi-closed, closed systems); housing systems (free-range, paddock, stable); social housing (individual, sex-separated, mixed groups); and self-assessed welfare knowledge (poor, moderate, good).

Self-assessed welfare knowledge was evaluated using a linear scale (1–10) and subsequently categorized into three groups: poor (scores 1–3), moderate (scores 4–6), and good (scores 7–10). This categorization was applied to facilitate the interpretation of the results and to allow comparison with similar descriptive studies.

Responses to open-ended questions were reviewed and grouped into thematic categories based on content similarity using a descriptive coding approach. The 1–10 linear scale used to assess economic viability was treated as an ordinal variable and applied to capture subjective perceptions of respondents in a structured manner. The scale was selected to allow respondents to express nuanced subjective perceptions.

In addition to descriptive statistics, Fisher’s exact tests were applied to examine associations between key categorical variables (e.g., knowledge × generation; fecal testing × purpose; vet access × location). A Kruskal–Wallis test was used to compare economic viability scores across subgroups. Ordinal logistic regression was used to identify predictors of self-assessed knowledge (poor/moderate/good), and binary logistic regression was used to identify predictors of post-treatment fecal examination. All analyses were performed in Python v.3.14.4 (scipy v.0.14.6, statsmodels v.0.14.6). Statistical significance was set at *p* < 0.05.

### 2.3. Ethical and Data Protection Considerations

Participation in the survey was voluntary, and respondents provided informed consent before completing it. All data were collected and processed anonymously, as the study involved only questionnaire-based data collection and did not involve animal experimentation, formal ethical approval was not required. The Scientific and Innovation Committee of the University of Veterinary Medicine, Budapest, approved the questionnaire.

## 3. Results

### 3.1. Demographic Characteristics of Respondents

**Figure 1:**
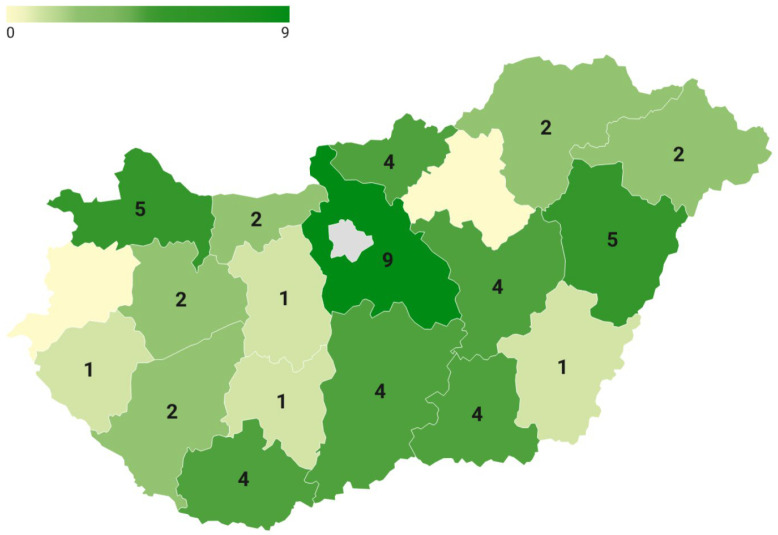
Spatial distribution of alpaca herds in Hungary based on survey responses. Color intensity and numbers in the regions represent the number of alpaca keepers per county. The pink area represents Budapest, the capital of Hungary.

The results should be interpreted in light of the methodological limitations described in the Discussion. A total of 53 Hungarian alpaca keepers completed the questionnaire between May and July 2023 (Figure 1).

The main demographic characteristics of the respondents are summarized in Table 1 and Table 2. The gender distribution of the respondents was balanced: 54.7% female (*n* = 29) and 45.3% male (*n* = 24) (Table 2). Generation X (44–58 years) represented the largest group (52.8%), followed by Generation Y (29–43 years; 30.2%). Generation Z (14–28 years) accounted for 7.5%, and respondents aged 59+ accounted for 9.4% (Table 1 and Table 2).

Alpacas are kept in urban areas (34.0%), small towns (37.7%), villages (17.0%), and farm or rural environments (11.3%). The highest concentration of herds was located in Central Hungary (Pest County). There were no respondents in Budapest (Figure 1). Most respondents (79.2%) had previous experience with animal keeping, including 13.2% who had kept camelids (llamas or camels; Table 1). In 64.2% of cases, animal care was performed by the entire family, while 26.4% employed permanent staff.

Regarding the duration of alpaca keeping, 75.5% started within the last five years, 22.6% between 6 and 15 years ago, and 1.9% more than 15 years ago. Gen X owners kept the majority of the alpacas (Table 2).

### 3.2. Herd Structure and Housing Characteristics

The total number of alpacas reported in the survey was 266. Huacaya alpacas accounted for 69.8% of herds, Suri alpacas accounted for 17.0%, and 13.2% reported mixed herds. Most alpacas were kept in year-round free-range systems with access to sheltered areas (67.9%). 17.0% reported semi-closed systems, and 7.5% used seasonal grazing systems. No respondent reported exclusively year-round closed housing. Mixed-sex housing was the most common system (81.1%), while ten farms separated males and females. Co-housed species included sheep and goats (57.7%), horses and poultry (30.8%), and deer, camels, or emus (11.5%).

### 3.3. Keeping and Breeding Purposes and Motivations

The primary purposes of alpaca farming were hobby (54.7%), supplementary income (32.1%), and animal-assisted services (3.8%). None of the respondents indicated alpaca keeping as their primary source of income.

**Figure 2:**
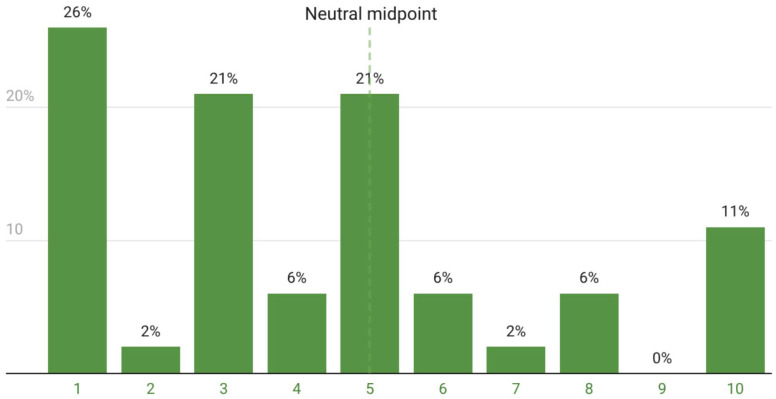
Perceived economic profitability of alpaca keeping in Hungary based on respondents’ evaluations (*n* = 53). Scores range from 1 (disadvantageous) to 10 (profitable). The vertical line indicates the neutral midpoint (score = 5).

Reported motivations included hobby and leisure (26.9%), exhibitions and petting zoos (19.0%), breeding and cria sales (12.5%), fiber production (11.5%), grazing and land management (8.7%), alpaca trekking (6.7%), event participation (5.8%), animal-assisted services (5.8%), and environmental education (2.9%). No respondent reported meat production as a purpose. Most respondents did not consider alpaca keeping to be economically efficient (Figure 2). When performing the Kruskal–Wallis test for economic viability, alpaca keeping was broadly perceived as economically unfavorable across all demographic categories (by generation: H = 1.24; *p* = 0.74) and by duration of keeping (H = 4.91; *p* = 0.086). A trend worth noting: longer-keeping owners gave higher scores (median 8.0 for >10 years vs. 3.0 for ≤5 years), but the sample is too small for certainty.

Regarding self-assessed knowledge about alpacas, 17.0% rated their expertise as poor, 43.4% as moderate, and 39.6% as good. The tested associations between generation, keeping duration, gender, previous livestock experience, and daily observations × knowledge were not significant (*p* > 0.05). When performing ordinal logistic regression on predictors of self-assessed knowledge, only one predictor was significant: respondents aged 59+ had lower odds of rating their knowledge as good compared to Gen X (OR = 0.09, 95% CI 0.01–0.69, *p* = 0.021). All other predictor effects (generation, duration, location, gender, previous livestock experience) were non-significant.

Regarding animal origin, 54.1% of respondents purchased alpacas from Hungarian breeders, 32.8% from abroad (mainly Germany and the Netherlands), and 17.0% from mixed sources. Among breeders, 86.0% used their own males for mating, 7.0% used external males, and 7.0% applied a combined strategy. Future herd expansion was planned by 67.9% of respondents: 51.0% through own breeding, 27.5% via domestic purchase, and 21.6% through foreign acquisition.

### 3.4. Welfare and Health Management

Daily observation of animals was reported by 84.9% of respondents, weekly by 5.7%, monthly by 5.7%, and 3.8% reported no regular observation. Regarding record-keeping, 20.6% did not maintain records; 15.1% relied solely on memory; and 50.9% recorded medical treatments, 28.3% pregnancies, 24.5% diseases, and 17.0% mortality events. Veterinary services were locally available for 79.2% of respondents, accessible only from distant locations for 11.3%, and difficult to access for 9.4%. However, 11.3% had never consulted a veterinarian. There was no significant association between the farm location and vet access (*p* > 0.05). The most commonly reported health problems included parasitic infections (45.0%), digestive and respiratory diseases (16.7%), injuries (13.3%), sudden death (10.0%), and lameness, abortion, or fleece-related problems (5.0% each). Post-mortem examination was requested in all cases by 13.2% of respondents, only in cases of unknown death by 20.8%, and never by 66.0%. The low frequency of post-mortem examinations and the lack of systematic health record-keeping may hinder early detection of infectious diseases and limit the ability to monitor disease occurrence at the herd level. This may have important epidemiological implications, including the potential for undiagnosed conditions and delayed intervention.

Antiparasitic treatments were applied every 2–6 months by 39.6%, annually by 26.4%, only when necessary by 22.6%, and every 1–2 months by 11.3%. Ivermectin was the most frequently used drug (73.6%), administered either intramuscularly or orally. Routine fecal examinations were performed by 45.3% of respondents, while 52.8% did not conduct laboratory testing. Treatment efficacy control was reported by 30.2%. Owners who had kept alpacas for longer were more likely to perform post-treatment fecal egg count monitoring. Among those keeping alpacas for >10 years, 80% performed fecal exams, compared to only 20% of those with ≤5 years of experience (*p* = 0.012). Other tested associations (generation and previous livestock experience) were not significant (*p* > 0.05). When performing binary logistic regression (predictors of fecal examination), one predictor was significant: keepers with >10 years of experience had higher odds of performing post-treatment fecal exams than those with ≤5 years of experience (OR = 13.91, 95% CI 1.16–167.31, *p* = 0.038). Self-assessed knowledge, gender, previous livestock experience, and generation were all non-significant predictors.

Hoof trimming was performed annually by 43.4% of respondents, semi-annually by 30.2%, every few months by 22.6%, and monthly by 1.9%. Hoof care was carried out by professional specialists (50.9%), veterinarians (18.9%), or the owners themselves (35.8%). Shearing was performed once annually in spring by 90.6%, at other times by 5.7%, twice annually by 1.9%, and never by 1.9%. Supplementary feeding was provided regularly to the entire herd by 64.2%, only to pregnant or weak animals by 20.8%, and not provided by 15.1%. The most common bedding type was a combination of straw and wood shavings (54.7%), while 24.5% used concrete flooring systems.

## 4. Discussion

The global spread of alpaca keeping beyond South America—and the shift in keeping objectives from fiber and pack use toward recreation, therapy, and hobby farming—has brought with it a new set of management and welfare challenges that differ markedly from the traditional pastoral context in which the species evolved [15].

Our survey, representing the first nationwide study, aimed to assess the current status of alpaca keeping in Hungary, including management practices, animal welfare conditions, and health-related issues. At the time of the survey, there was no official obligation to register the alpacas in Hungary. It is not yet possible to register alpacas in the ENAR—the National Unified Registration and Identification System for Livestock—but they can be voluntarily registered in the Small Animal Registry of the Hungarian Veterinary Chamber [19]. Consequently, not all animals are individually identified and registered [19]; thus, the total number of alpacas in Hungary is still unknown. Similarly, it is not known how many farms keep alpacas in Hungary. Some farmers register their animals with the Hungarian Veterinary Chamber, while others register them with Llama and Alpaca Registries Europe (https://lareu.org/index_EN.html, accessed on the 20 February 2026). There is no national alpaca breeding association; therefore, some farms are registered abroad, for instance, with the Austrian Alpaca Association. According to their communication, there are seven Hungarian owners and 83 Hungarian alpacas registered in the LAREU registry. The Hungarian Veterinary Chamber communicates similar numbers. When preparing the survey, we estimated that there were 300 animals across around 20 farms at the start of the study. Surprisingly, 53 farms reported keeping at least one alpaca, and the total number of animals in our survey was 266, which is relatively small compared to populations reported in other countries. This discrepancy suggests that initial estimates of the alpaca population in Hungary were likely underestimated, highlighting the absence of reliable baseline data. Therefore, the findings should be interpreted as descriptive insights rather than as fully representative of the national population. Although the sample likely captures a substantial proportion of alpaca keepers in Hungary, the lack of centralized registry data prevents a full assessment of representativeness. These findings underline the need for establishing a national registry system to improve data reliability in the future.

Similar to other European countries [10,11], alpaca keeping in Hungary is a young, dynamically developing sector with considerable economic, recreational, and therapeutic potential. The motivational patterns and welfare-related attitudes observed among respondents are largely consistent with trends reported in Western European studies [8,10]. In Hungary, alpaca keeping is primarily driven by emotional attachment, leisure activities, and a nature-oriented lifestyle rather than intensive production. While this approach supports welfare-oriented practices, it may also limit the depth of professional knowledge, as reflected in respondents’ self-assessments. The depth of knowledge about alpacas was self-assessed as moderate or poor by more than 60% of respondents, consistent with findings from similar studies in other European countries [10,11]. It indicates that species-specific management knowledge has not yet been fully integrated into general husbandry practices. Respondents aged 59+ had significantly lower odds of rating their knowledge as good than Gen X respondents, suggesting that older respondents may be less engaged with online information communities where alpaca knowledge circulates.

According to the international literature, alpaca welfare is closely associated with housing systems and owners’ observation practices [4,11]. Common welfare challenges include inadequate nutrition, delayed recognition of parasitic infections, and behavioral disorders such as Berserk Male Syndrome [5]. More than 80% of the owners in our study reported daily observation of their animals, a strong welfare indicator that facilitates early disease detection and recognition of behavioral changes. However, reliance on self-reported data may introduce bias, as respondents may overestimate certain practices, such as the frequency of daily animal observation. Preventive health management practices, including routine fecal examinations, quarantine protocols, and systematic health record keeping, were implemented by only a minority of farms, suggesting that many owners still rely primarily on experiential knowledge rather than structured, veterinarian-guided management strategies.

One of the most important welfare-related findings of this survey is the widespread use of social housing systems, including group and mixed-sex housing, which align with alpacas’ natural behavior and are in line with other studies [10]. As a highly social species, alpacas are sensitive to isolation, which may induce stress and behavioral disorders such as Berserk Male Syndrome, as reported in previous studies [5]. Group housing, adequate space allowance, and visual contact with conspecifics therefore play a crucial role in maintaining optimal welfare conditions.

Further improvement is required in nutrition and parasite control. Although 64% of respondents regularly provided dietary supplements, supplementation practices were often empirical and rarely based on laboratory analyses. Ivermectin was the most commonly used antiparasitic agent, consistent with international findings indicating its widespread use in New World camelids (including alpacas, llamas, guanacos, and vicuñas) [21,22]. Based on our findings, the relatively low frequency of routine fecal examinations (45.3%) and treatment efficacy control (30.2%), combined with the widespread use of ivermectin, indicates a tendency toward preventive treatment without adequate diagnostic support. This practice may increase the risk of undetected parasitic infections and contribute to the development of antiparasitic resistance. These findings are consistent with reports from other European countries, where insufficient diagnostic-based parasite control has been identified as a common challenge in alpaca husbandry. While ivermectin-based treatments are generally effective, excessive or inappropriate use may contribute primarily to drug resistance [23,24] and adverse health effects, including toxicity, dermatological reactions, and increased stress. A German study examined 538 alpacas from 27 farms and found that fenbendazole treatment reduced strongyle egg excretion by only 45%, with most farms showing an inadequate treatment response. The authors conclude that monitoring treatment efficacy and implementing adapted deworming strategies should be applied in European alpaca herds [25]. Therefore, regular fecal egg counts and targeted treatment strategies should be encouraged to slow the development of anthelmintic resistance. The duration of alpaca keeping was the strongest predictor of evidence-based parasite management in our study, as experienced owners are significantly more likely to perform post-treatment fecal monitoring. Many owners rely on experiential rather than structured knowledge, and education efforts should be particularly targeted at newer keepers. Raising awareness of animal health issues and providing appropriate education for owners are, therefore, particularly important in countries where alpaca keeping is relatively new. Recent studies have highlighted that camelids’ sensitive diagnostic characteristics and species-specific management requirements often pose challenges for novice keepers, potentially resulting in welfare risks in the absence of proper training [26].

The provision of sheltered resting areas and access to outdoor enclosures represents another important welfare indicator. Approximately 68% of farms ensured year-round access to pasture, which may support welfare under appropriate management conditions. However, the present study did not assess pasture quality, stocking density, or seasonal variations in forage availability, which may influence the actual welfare outcomes associated with pasture access. In addition, the use of concrete flooring systems on some farms (24.5%) may increase the risk of lameness and injuries if not properly managed. The absence of exclusively indoor housing systems suggests that most farms provide environments that may meet alpacas’ physiological needs [4].

Shearing and hoof care frequency also play a key role in welfare management. More than 90% of respondents reported annual shearing, consistent with animal welfare recommendations and previous studies [5,10]. However, specific European guidelines for alpaca shearing and hoof care are limited, and the present study did not assess compliance with standardized protocols. Insufficient shearing may result in overheating and skin disorders, whereas excessive shearing can increase stress levels and impair thermoregulation. In addition, no analysis was conducted to examine potential associations between care frequency and housing conditions, which may influence welfare outcomes. Therefore, these findings should be interpreted in a descriptive rather than normative context.

An important outcome of the present study is the identification of a strong community network among Hungarian alpaca keepers. Approximately 70% of respondents obtained information from veterinarians, 55% from other breeders, and 49% from online community groups. This horizontal knowledge-sharing structure may facilitate the dissemination of good welfare practices. However, establishing national guidelines defining minimum standards for camelid husbandry—including housing conditions, space requirements, thermal comfort, feeding, and health management—would further strengthen professional standards. Establishing a national alpaca association or formal extension programs could further support the standardization and dissemination of best management practices.

Overall, current alpaca-keeping practices in Hungary demonstrate several favorable welfare-related characteristics. Nevertheless, further development is needed in systematic record-keeping, evidence-based preventive medicine, and structured education. Given the openness of the national breeder community and increasing international collaboration, the welfare standards of alpaca keeping in Hungary are likely to continue improving in the coming years.

## 5. Limitations

This study has several limitations that should be considered when interpreting the results. First, participation in the survey was voluntary, which may have introduced self-selection bias, as more engaged or motivated alpaca keepers were more likely to respond. Second, the data were based on self-reported information, which may not always accurately reflect actual management practices. Furthermore, reported disease data were not diagnostically confirmed and should therefore be interpreted with caution. In addition, self-assessed knowledge levels may not accurately reflect actual expertise. Third, although the sample size (*n* = 53) is reasonable given the estimated size of the alpaca-keeping sector in Hungary, it may not fully capture the diversity of existing practices. Finally, the survey was conducted in 2023, and management practices may have changed since then.

## 6. Conclusions

This study provides the first nationwide overview of alpaca keeping in Hungary, covering owner demographics, husbandry practices, health management, and welfare-related attitudes. The Hungarian alpaca sector is small but growing rapidly. Most herds are kept on small, family-based farms with strong daily monitoring and close human–animal bonds—conditions that may support animal welfare. Free-range housing with adequate space and shelter is common, and a high proportion of owners report daily observation of their animals. However, several important gaps were identified. Biosecurity practices remain limited: only a minority of owners use laboratory diagnostics, quarantine measures, and systematic health record-keeping. The absence of mandatory individual identification for New World camelids in Hungary further hinders disease traceability and control. The geographical dispersal of herds, combined with limited biosecurity awareness, may increase the risk of infectious disease transmission and antimicrobial resistance—carrying implications for animal health and disease control at the herd level. These conclusions are based on descriptive data and should be interpreted within the limitations of the study design. Alpaca keeping may also be considered within the broader One Health framework; however, this study did not directly assess the risks related to human or environmental health. Strengthening owner education, developing national husbandry guidelines, improving data transparency, and promoting professional training are key priorities for the long-term sustainable and ethical development of alpaca keeping in Hungary.

## Figures and Tables

**Table 1:** Demographic characteristics of alpaca keepers participating in the survey (*n* = 53). Values represent the number and % of total respondents (*n* = 53). Subcategories (e.g., camelids) refer to specific types of previous animal-keeping experience.

	Characteristics	Number of Respondents (*n*)	Percentage (%)
Age	Gen Z (14–28)	4	7.5
	Gen Y (29–43)	16	30.2
	Gen X (44–58)	28	52.8
	59+	5	9.5
Gender	Male	24	45.3
	Female	29	54.7
Location type	Urban	18	34.0
	Small town	20	37.7
	Village	9	17.0
	Farm/rural	6	11.3
Previous experience with animal keeping	Yes	42	79.2
	of which camelids	7	13.2
	No	11	20.8

**Table 2:** Distribution of alpaca population by respondent characteristics. Values represent the number and % of total alpacas (*n* = 266).

	Characteristics	Number of Alpacas (*n*)	Percentage (%)
Age	Gen Z (14–28)	24	9.1
	Gen Y (29–43)	75	28.1
	Gen X (44–58)	141	53.1
	59+	26	9.7
Gender	Male	114	42.9
	Female	152	57.1

## Data Availability

The data presented in this study are available upon request.

## Supplementary Materials

The following supporting information can be downloaded at https://www.mdpi.com/article/10.3390/ani16081209/s1. The English version of the anonymous online questionnaire is available as a Supplementary File.
