# Supplementary material for: Alpaca Keeping in Hungary: The First Nationwide Survey
Source: Animals (Basel). 2026 Apr 16;16(8):1209. doi: 10.3390/ani16081209 (PMC13113044; doi:10.3390/ani16081209)
Supplement: Supplementary file 1 [file animals-16-01209-s001.zip › animals-4191694-supplementary.pdf]

Questionnaire Used in the Survey (English Version)

Alpaca Keeping – Questionnaire

Dear Alpaca Owner,

My name is Viktória Láng, and I am a student at the University of Veterinary Medicine. My main area of interest is camelids, especially alpacas. In addition to my university studies, I am also studying in England, focusing on health-related issues concerning alpacas. As part of my Scientific Students' Association (TDK – Scientific Students' Conference) research, I would like to conduct a survey among alpaca owners on this topic. By completing this questionnaire, you will help me gain a comprehensive understanding of the needs, objectives, health status, approximate population size, and geographical distribution of alpaca owners in Hungary.

I kindly ask you to support my work by completing this questionnaire, which takes approximately 15 minutes. Participation in this study is entirely voluntary and anonymous. All responses will be used solely for scientific purposes and will be handled confidentially in accordance with applicable data protection regulations. Even in the case of future publication—possibly in a scientific journal—no individual data will be disclosed to third parties. Results will be presented only in aggregated, processed, and non-identifiable form.

If you have any questions regarding the questionnaire or would like to learn about the results later, please feel free to contact me at: tdk.alpaka@gmail.com.

Thank you very much for your valuable contribution to the success of my research.

1. Age
☐ Under 18
☐ 18–25
☐ 26–35
☐ 36–45
☐ 46–55
☐ 56–65
☐ Over 65

2. Gender
☐ Female
☐ Male
☐ Prefer not to say

3. In what form do you keep alpacas?
☐ Primary source of income (main livelihood)
☐ Supplementary income (in addition to other activities)
☐ Hobby/family tradition
☐ Companion animals
☐ Other: ____________________

4. For what purpose do you keep alpacas?
☐ Breeding

☐ Meat production
☐ Fiber production
☐ Open farm/zoo for visitors
☐ Event participation (e.g., weddings, photo sessions)
☐ Therapeutic purposes (e.g., child development)
☐ Hobby
☐ Pasture maintenance/grazing
☐ Tourism (e.g., alpaca camping, guesthouse, apartment)
☐ Alpaca trekking/walking for visitors
☐ Economic profit
☐ Environmental awareness
☐ Other: ____________________

5. Do you have assistance in caring for your alpacas?
☐ No, I take care of them alone
☐ Yes, with my family
☐ Yes, seasonal employees help
☐ Yes, permanent employees help
☐ Only when I am away (vacation substitute)

6. Where is your alpaca farm located?
☐ Urban residential area
☐ Village residential area
☐ Small town residential area
☐ Urban outskirts
☐ Village outskirts
☐ Small town outskirts
☐ Remote rural area (>5 km from settlement)

7. In which county do you keep alpacas?
☐ Bács-Kiskun
☐ Baranya
☐ Békés
☐ Borsod-Abaúj-Zemplén
☐ Csongrád-Csanád
☐ Fejér
☐ Győr-Moson-Sopron
☐ Hajdú-Bihar
☐ Heves
☐ Jász-Nagykun-Szolnok
☐ Komárom-Esztergom
☐ Nógrád
☐ Pest
☐ Somogy
☐ Szabolcs-Szatmár-Bereg
☐ Tolna
☐ Vas
☐ Veszprém
☐ Zala

8. Which settlement does your farm belong to?

____

9. Did you keep large livestock before alpacas? (e.g., cattle, sheep, goats, horses, pigs)
☐ Yes, cattle
☐ Yes, pigs
☐ Yes, goats
☐ Yes, sheep
☐ Yes, horses
☐ Yes, llamas/camels
☐ Yes, other livestock
☐ No, I had no previous experience

10. Do you currently keep other large livestock besides alpacas?
☐ Yes, cattle
☐ Yes, pigs
☐ Yes, goats
☐ Yes, sheep
☐ Yes, horses
☐ Yes, llamas/camels
☐ Yes, other livestock
☐ No, only alpacas

11. How long have you been keeping alpacas?
☐ Less than 1 year
☐ 1 year
☐ 2–5 years
☐ 6–10 years
☐ 11–15 years
☐ More than 15 years

12. What sex of alpacas do you keep?
☐ Only females
☐ Only males
☐ Mixed (males and females)

13. How many adult alpacas do you currently keep (over 1.5 years)?
☐ 1
☐ 2
☐ 3
☐ 4
☐ 5
☐ 6–10
☐ 11–15
☐ 15+

14. How many crias/young alpacas (under 1.5 years) do you currently keep?
☐ 0
☐ 1
☐ 2
☐ 3
☐ 4
☐ 5
☐ 5+

15. How many adult males (over 1.5 years) do you currently keep?
☐ 0
☐ 1
☐ 2
☐ 3
☐ 4
☐ 5
☐ 5+

16. Which alpaca breed do you keep?
☐ Suri
☐ Huacaya
☐ Both

17. From where did you obtain your initial alpacas?
☐ Hungarian breeder
☐ Foreign breeder
☐ Domestic livestock markets
☐ Foreign livestock markets
☐ Other: ____________________

18. Do you plan to expand your herd? If yes, in what way?
☐ I do not plan to expand
☐ Through own breeding
☐ By purchasing animals domestically
☐ By purchasing animals from abroad
☐ Other: ____________________

19. What type of breeding male do you use for mating?
☐ Only my own male
☐ Male from another domestic breeding farm
☐ Male from a foreign breeding farm
☐ Other: ____________________

20. Where are the alpacas housed?
☐ Kept only in enclosed barns/buildings
☐ Kept indoors without outdoor access in winter; on pasture in summer
☐ Kept indoors with outdoor access in winter; on pasture in summer
☐ Kept indoors year-round with a maximum 100 m^2 outdoor enclosure
☐ Kept indoors year-round with a maximum 1000 m^2 outdoor enclosure
☐ Kept outdoors year-round, with shelter available in bad weather
☐ Other: ____________________

21. Housing management of alpacas
☐ Males and females are kept together
☐ Males and females are kept separately
☐ Alpacas share pasture/enclosure/buildings with other livestock
☐ Alpacas are kept separately from other livestock
☐ Only the pasture/enclosure is shared; housing is separate
☐ No other livestock are kept with alpacas

22. If alpacas share facilities with other livestock, which animals are kept together with them?
☐ None
☐ Horses
☐ Cattle
☐ Sheep
☐ Goats
☐ Pigs
☐ Poultry
☐ Llamas and/or camels
☐ Other: ____________________

23. Where do you obtain information related to animal health and diseases?
☐ Internet sources
☐ Printed professional books
☐ Digital books
☐ Printed professional journals
☐ Digital journals
☐ Alpaca groups (e.g., Facebook)
☐ Other alpaca breeders
☐ Veterinarians
☐ Feed company representatives
☐ Other: ____________________

24. How did you get the idea to keep alpacas?
☐ I read about them and became interested
☐ I saw them at a fair and became interested
☐ I saw them at a friend's or acquaintance's farm
☐ I encountered them at an animal/alpaca farm
☐ Other: ____________________

25. In your opinion, how profitable and worthwhile is alpaca farming in Hungary?
1 2 3 4 5 6 7 8 9 10
Not profitable ⟶ High potential and worth developing

26. How do you use alpaca fleece?
☐ As raw, unprocessed material
☐ Partly processed (e.g., yarn)
☐ Sold as finished products in own shop
☐ Sold as finished products to retailers
☐ Other: ____________________

27. If you produce products, what do you make from alpaca fleece?
____

28. How often do you monitor your animals and their health status?
☐ Daily
☐ Weekly
☐ Every two weeks
☐ Monthly
☐ I do not monitor them regularly

29. Do you keep any records related to your animals?
☐ Diseases
☐ Mortality and causes of death
☐ Medical treatments
☐ Breeding and pregnancy
☐ Fleece quality
☐ Income and expenses
☐ Animal movements (sales, purchases)
☐ I keep records only mentally
☐ I do not keep any records
☐ Other: ____________________

30. What happens to alpacas returning to the farm or newly introduced animals? (Quarantine)
☐ Isolated for 3–5 days
☐ Isolated for 2–3 weeks
☐ Isolated for 4 weeks
☐ Not isolated; immediately reintroduced
☐ Other: ____________________

31. Is there a veterinarian nearby who can assist with alpaca-related issues?
☐ Yes
☐ No
☐ A veterinarian travels from a distant location

32. When did you last consult a veterinarian regarding your alpacas?
☐ Never
☐ Within the past year
☐ 1–2 years ago
☐ 3–5 years ago
☐ More than five years ago
☐ Only for certification/transport documentation

33. If you do not consult a veterinarian, why not?

____

34. What do you do if your alpacas become ill?
☐ My alpacas never get sick
☐ I do nothing
☐ I treat them based on my own experience
☐ I ask another breeder for help
☐ I consult a veterinarian
☐ I consult a feed company representative
☐ Other: ____________________

35. Have you experienced behavioral problems, and if so, how did you handle them?
☐ No
☐ Yes, I consulted a veterinarian
☐ Yes, I consulted another breeder
☐ Yes, I searched for solutions online
☐ Other: ____________________

36. If you experienced behavioral problems, what were they?

____

37. What are the most common diseases observed in your herd?
☐ Internal parasites
☐ External parasites (e.g., mange)
☐ Diarrhea
☐ Respiratory diseases
☐ Abortion
☐ Mastitis
☐ Foot disorders (lameness)
☐ Skin injuries
☐ Fleece abnormalities
☐ Sudden death
☐ Other: ______________________

38. Are pathological examinations (necropsies) performed?
☐ Yes, regularly, even if the cause of death is clear
☐ Yes, if the cause of death is unclear
☐ No

39. What antiparasitic treatments do you use?
☐ Injectable preparations (e.g., ivermectin, deltamethrin)
☐ Pour-on formulations
☐ Bathing
☐ Oral paste treatments
☐ Herbal baths and herbal feeding
☐ Other: ______________________

40. Which antiparasitic agent(s) or active substance(s) do you use most frequently?

____

41. How frequently are antiparasitic treatments administered?
☐ Every 5–6 weeks
☐ Every 7–8 weeks
☐ Every 2–6 months
☐ Every 7–12 months
☐ No regular treatment, only as needed

42. What dosage is used for anthelmintic treatments?
☐ Recommended sheep dosage
☐ 1.5 times the recommended sheep dosage
☐ Twice the recommended sheep dosage
☐ Three times the recommended sheep dosage
☐ Four times the recommended sheep dosage
☐ Other: ______________________

43. On whose recommendation do you determine the dosage?
☐ Veterinarian
☐ Information from social media
☐ Experience of other breeders
☐ Professional journals/magazines

☐ Personal judgment
☐ Internet sources
☐ Other: ____________________

44. Have fecal or skin examinations for parasites been performed?
☐ Yes
☐ Yes, regularly
☐ No
☐ Other: ____________________

45. Is follow-up fecal examination performed after deworming?
☐ Yes
☐ No

46. After deworming, is feces removed for 1–2 days?
☐ Yes, from the barn/building only
☐ Yes, from the barn/building and the enclosure
☐ Yes, from the enclosure only
☐ No

47. How often are hooves trimmed?
☐ Monthly
☐ Every few months (every 3–4 months)
☐ Approximately every six months
☐ Once a year
☐ Never

48. Who performs hoof trimming?
☐ The owner/keeper
☐ External professional
☐ Veterinarian
☐ Other: ____________________

49. Who performs dental trimming?
☐ The owner/keeper
☐ External professional
☐ Veterinarian
☐ Other: ____________________

50. Are footbaths used occasionally?
☐ Yes, in case of disease
☐ Yes, regularly for prevention
☐ Not used
☐ I do not know what a footbath is
☐ Other: ____________________

51. How would you rate your knowledge of alpaca husbandry?
1 2 3 4 5 6 7 8 9 10
Very low ⟶ Very high

52. How often are alpacas sheared?
☐ Never

☐ Once a year, in spring
☐ Once a year, not in spring
☐ Twice a year

53. Are nutritional and/or vitamin supplements used?
☐ No
☐ Yes, for pregnant animals
☐ Yes, during mating
☐ Yes, during the breeding cycle
☐ Yes, regularly for all animals
☐ Yes, for sick/weakened animals
☐ Yes, in winter/cold periods
☐ Other: ____________________

54. What type of flooring is used in the barn/building?
☐ Concrete with bedding
☐ Sand with bedding
☐ Brick with bedding
☐ Resin flooring with bedding
☐ Other: ____________________

55. How often are females bred?
☐ Once a year
☐ Every two years
☐ Every three years
☐ Never; animals are purchased instead
☐ Other: ____________________

Acknowledgement

Dear Alpaca Keeper,

Thank you very much for taking the time and effort to complete this questionnaire. Although it may have seemed like a large number of questions over the past few minutes, their purpose was to provide a comprehensive overview of the current state of alpaca husbandry in Hungary—a field for which very limited information is available not only domestically but also internationally.
By participating in this survey, you have contributed not only to my future professional development as a veterinarian, but also to the creation of an important summary for alpaca keepers regarding the current situation of alpaca husbandry in Hungary. This overview may serve as a valuable basis for further improvements and development in several areas.

If there is sufficient interest, the aggregated and analyzed results of the survey will be made publicly available, including publication in professional journals. For further information, please contact: tdk.alpaka@gmail.com.

Sincerely,
Viktória Láng
